# The impact of COVID-19 related regulations and restrictions on mobility and potential for sustained climate mitigation across the Netherlands, Sweden and the UK: a data-based commentary

**DOI:** 10.14324/111.444/ucloe.000032

**Published:** 2022-02-24

**Authors:** Elizabeth Corker, Kaloyan Mitev, Astrid Nilsson Lewis, Milan Tamis, Thijs Bouman, Stefan Holmlid, Fiona Lambe, Susan Michie, Matthew Osborne, Reint Jan Renes, Linda Steg, Lorraine Whitmarsh

**Affiliations:** 1 Centre for Behaviour Change, Department of Clinical, Educational & Health Psychology, University College London, London, UK; 2 Department of Psychology, University of Bath, Bath, UK; 3 Stockholm Environment Institute, Stockholm, Sweden; 4 Research Group Psychology for Sustainable Cities, Amsterdam Research Institute for Societal Innovation, Amsterdam University of Applied Sciences, Amsterdam, The Netherlands; 5 Department of Psychology, Faculty of Behavioural and Social Sciences, University of Groningen, Groningen, The Netherlands; 6 Department of Computer and Information Science, Linköping University, Linköping, Sweden; 7 Centre for Climate Change and Social Transformations (CAST), Linköping, Sweden

**Keywords:** climate change, behaviour change, COM-B, moment of change, COVID-19, people and their environment

## Abstract

Human behaviour change is necessary to meet targets set by the Paris Agreement to mitigate climate change. Restrictions and regulations put in place globally to mitigate the spread of COVID-19 during 2020 have had a substantial impact on everyday life, including many carbon-intensive behaviours such as transportation. Changes to transportation behaviour may reduce carbon emissions. Behaviour change theory can offer perspective on the drivers and influences of behaviour and shape recommendations for how policy-makers can capitalise on any observed behaviour changes that may mitigate climate change. For this commentary, we aimed to describe changes in data relating to transportation behaviours concerning working from home during the COVID-19 pandemic across the Netherlands, Sweden and the UK. We display these identified changes in a concept map, suggesting links between the changes in behaviour and levels of carbon emissions. We consider these changes in relation to a comprehensive and easy to understand model of behaviour, the Opportunity, Motivation Behaviour (COM-B) model, to understand the capabilities, opportunities and behaviours related to the observed behaviour changes and potential policy to mitigate climate change. There is now an opportunity for policy-makers to increase the likelihood of maintaining pro-environmental behaviour changes by providing opportunities, improving capabilities and maintaining motivation for these behaviours.

## Introduction

Researchers expected that the large-scale behaviour changes required to mitigate the impact of COVID-19 were improbable [1]. However, responses to the COVID-19 pandemic have resulted in far-reaching behavioural changes across nations in everyday living, such as mobility and working practices [2,3]. Furthermore, these often thought to be improbable behaviour changes may impact carbon emissions. For example, increased working from home may reduce carbon emissions from commuters, whilst restricting public transport use may increase private car use and carbon emissions. The pandemic-related restrictions and guidance across Europe presented an opportunity to describe real-time changes in behaviour related to climate change. Examples of measured impacts due to these changes are improved air quality [4–6], noise reduction in inner cities [7] and carbon emissions [3].

To build on the window of opportunity, the UK Foreign, Commonwealth and Development Office convened the European Behaviour Change Network (EBCN), comprising experts in behavioural science, health and environmental psychology, service design and engineering from the Netherlands, Sweden and the UK. The EBCN initiated a project to identify changes in citizens’ behaviours during the COVID-19 pandemic that might influence climate mitigation and help stay below a 2°C increase in climate change.

Long-term changes in climate variability resulting from human activity and leading to climate change [8] are one of several major problems faced by citizens, governments and policy-makers globally [9].

Changes in human behaviour are necessary to achieve climate targets and behavioural sciences can contribute to tackling climate change by understanding current behaviours and identifying ways in which behaviours could change [10–12]. Decades of work within the behaviour change field has provided insights into how to develop and deliver behaviour change interventions [12–18]. These insights remain underexploited in government policies to tackle climate change, which often remain largely technology-focused [19].

Governments are adopting targets to cut carbon emissions; for example, the UK and Sweden aim to produce ‘net zero’ emissions by 2050 and 2045, respectively, while the Netherlands’ target is a 49% reduction in emissions by 2030 and 95% by 2050. In 2020, many citizens across Europe were advised or instructed to ‘work from home’ where possible and to limit interactions between people to reduce COVID-19 infection transmission [20,21]. Working from home reduces the use of carbon-intensive transport [22]. Globally, the transport sector is estimated to contribute 14% of annual emissions, including non-carbon dioxide (CO_2_) gases and about a quarter of CO_2_ emissions, making transportation a sector with high climate mitigation potential [10]. Nations with highly developed economies produce the most carbon emissions, highlighting global inequalities and disparities [23]. Pro-environmental behaviour changes within countries with highly developed economies have the potential to impact climate mitigation [23].

Periods of disruption may offer unique ‘moments of change’: they can break behavioural patterns and routines due to a change in personal, social or professional circumstances [24]. Moments of change offer opportunities to intervene and reshape behavioural practices [25]. A moment of change may be an opportunity to promote pro-environmental behaviours. The COVID-19 pandemic, viewed as a moment of change, may represent a window of opportunity for policy-makers to initiate pro-environmental behaviour changes amongst citizens. Additionally, policy-makers may provide infrastructure and guidance to increase the likelihood that pro-environmental behaviour changes are maintained long-term.

Here, we describe changes in citizens’ transport use behaviours influenced by work-from-home policies during the COVID-19 pandemic. We then consider these changes within a behaviour science framework and identify their potential impact on climate change and policy opportunities for maintaining pro-environmental behaviour change and reversing behaviour change with an environmental cost. Our focus is on the following questions:

1. What transport use-related behaviour changes have been made by citizens during the 2020 COVID-19 pandemic in the Netherlands, Sweden and the UK?
2. What is the likely impact of any behaviour changes on climate change?
3. How can pro-environmental changes be sustained long-term, and how can changes with an environmental cost be reversed?

We address these questions using data from 28 sources: 12 from the Netherlands, nine from Sweden and seven from the UK. The sources reflected data collected from before COVID-19 restrictions were in place to December 2020. Data included traffic counts, mobile GPS data, sales data and self-report survey data from freely available government and commercial sources. We used multiple sources to triangulate data. We present the following as an illustration of transport-related behaviour changes across the Netherlands, Sweden and the UK. For a description of the methods, see Appendix 1, and for a list of the data sources used for each country, see Appendix 2. We do not compare data between countries due to differences in COVID-19 related measures, cultures and policy agendas, along with varied data sources used in this research.

## Changes in transport use made by citizens during the COVID-19 pandemic

During the COVID-19 pandemic in 2020, varying restrictions, policies and recommendations relating to behaviour were in place. For an overview and timeline of the decisions made in the Netherlands, Sweden and the UK, see Appendix 3.

Globally, in April 2020, COVID-19 associated restrictions were predicted to decrease public transport use and car traffic related to daily activities such as commuting to work, socialising and shopping, along with a global reduction of carbon emissions [22,23]. Globally, a 50–75% reduction in road traffic was seen during lockdown restrictions [26]. Travel restrictions may reduce well-being [27,28]; however, they also provide physical and psychological opportunities to adopt transportation behaviours with less environmental impact [29]. Similarly, changes made to working from home could also lead to a decrease in the consumption of fossil energy through decreasing transportation demand [30]. Here, we bring together insights from diverse data sources to examine changes in behaviour across three European countries that reflect highly developed economies with different transport demand profiles and divergent policy responses to COVID-19. Total energy consumption encompasses more than individual transport behaviours. A global drop in energy demand was reported in 2020 and global energy-related carbon emissions were reduced by 5.8% compared to 2019, representing the largest annual decline since the Second World War, and suggesting that the reduction in energy use for transportation did not result in a shift in demand for energy use in domestic settings [31]. These global trends were also reported in the UK and Sweden [32,33].

## Netherlands data

The number of Dutch citizens working from home almost doubled, from 37% to 66%, between March 2020 and May/June 2020, with some citizens intending to work from home more often after COVID-19 related restrictions end (ranging from 26% to 45%) [34–37].

During March 2020, public transport use decreased, with Translink reporting 41–87% less public transport check-ins in 2020 compared to the same week in the previous year [34,38–42]. Dutch citizens opted for use of private cars (between 41% and 60%), bikes (between 14% and 57%), and walking (between 17% and 38%) instead, with proportions varying based on mode of public transport previously used [34,38–40,43]. Additionally, a 30% increase in occupancy rate and a 15% increase in number of rides from car-sharing services since May 2020 has been reported [44]. Data from June/July, September and November 2020 indicate that Dutch citizens biked and walked longer distances than before the COVID-19 restrictions [34,38,39]. Fifty-two percent of citizens reported intentions to continue biking instead of taking public transport once COVID-19 restrictions end [34,39].

Commuters travelling by public transport (60% by train, 35% by bus, tram or metro) reported avoiding rush hours [34,41,42]. Twenty-five percent of train travellers reported intentions to avoid rush hours after COVID-19 related restrictions end [36].

The sales of electric vehicles^1^ (plug-in hybrid vehicles and fully electric vehicles) increased by 40% in the period from January until October 2020 compared to the same period in 2019 [45–52]. While internal combustion engine car sales decreased by 23% from January to October 2020 compared to the same period in 2019, an increase in car purchase intention was reported, from 56% in May 2020 to 64% in September 2020 [47,51,53]. Thirty-four percent of people who reported using their car instead of public transport during COVID-19 related restrictions expected to use their car more often after COVID-19 restrictions end [34].

## Swedish data

Between February and April 2020, the proportion of people working remotely full-time increased from 2% to 32% [54]. Additionally, about 90% of survey respondents reported a desire to continue working from home, at least 1 day per week, when they have the option to return to their offices [54,55].

Overall, a significant decline in mobility was observed during the pandemic, suggesting that Swedes followed the Public Health Agency’s recommendations and worked from home to a great extent. Data from mobile phone densities demonstrated that people stayed more in residential areas during the day [56]. Additionally, the distance citizens moved from their homes over a day decreased by 38% between January and March 2020 [56]. Moreover, monitoring of urban noise patterns in central Stockholm showed a significant reduction in noise levels after the recommendation of working from home, confirming the mobility decline [7].

A 10–30% decrease in car traffic during weekdays and a 30–40% decrease on weekends between March and May 2020 was observed [57]. Furthermore, reports show a significant reduction in particulates measured on busy streets in Stockholm [6]. As car traffic decreased, so did car sales. The Swedish industry organisation for manufacturers and importers of cars recorded a decrease in private car sales of 18% in 2020 compared to 2019. Of car sales made, electric vehicles accounted for 32.2% compared to 11.3% in 2019 [58].

In terms of active transport, municipal data showed a decrease in pedestrian flows in the inner city of Stockholm; and a slightly lower decrease in the outer city [59]. Concerning biking, the municipality recorded a modest decrease in bike use in Stockholm on weekdays and an increase (30–100% between mid-March and June 2020) on weekends, suggesting citizens were using bikes during their leisure time [59]. A survey conducted in April 2020 found that 28.6% of respondents reported walking and biking more since the pandemic started, mainly to avoid public transport [60]. Data from ticket validations, sales and passenger counts between February and June 2020 shows a decrease of 40–60% in public transport use across the three most populated regions in Sweden compared to the same time in 2019.

## UK data

The proportion of people working from home increased in the UK from 11.4% before the pandemic to 36.2% during the first lockdown, decreasing slightly to 30.7% in November 2020 [61]. Additionally, 30.6% of people reported a desire to continue working from home after the restrictions related to COVID-19 are lifted [61].

Overall, there was a decrease in road traffic: 50–70% during the first national lockdown (March–May 2020) and 30–40% in October 2020, compared to pre-pandemic levels [62]. Levels of nitrogen dioxide (NO_2_) over London decreased by around 31% [4]. New car registrations declined by 29.4% compared to 2019 [63].

The use of public transport declined in the UK. Data indicated train use was down by 95% in the first lockdown compared to the same period in 2019 and 75% during the second national lockdown [62]. Ticketer, a system for tracking bus use, recorded an 80–85% decrease from March to June 2020 and a 55–65% decrease from November 2020 compared to January 2020 [62]. These data are consistent with data recording the number of check-ins at public Wi-Fi hotspots, which indicated a reduction of around 70% in public transport use weekly during the month of October 2020 [64].

Data from Transport for London showed tube journeys decreased by 94% in April and May 2020, and by 64% in mid-August to mid-October, compared to the same periods in 2019 [65]. Bus use in London was down by 80% in April–May 2020, and 42% in October 2020 [65] compared to data for the same time points in 2019. These data are consistent with data from Citymapper showing a reduction of transport use in London by 80–90% in April and May 2020 and 40–50% in November 2020 compared to 2019 [66].

Cycling trips increased by 100–200% during April–June 2020 and by 50–80% in July–October 2020 compared to March 2020, as reported by data from mobile phone network provider O2 [62]. London specific data from the Santander Cycle Hire Scheme indicated a mixed impact with a decrease in bike hires of 34% in April 2020, an increase of 15% in May 2020, and an increase of 18% in September 2020 when compared to the same periods in 2019 [65].

Sales of electric vehicles in 2020 increased by 185.9% and 91.2%, respectively, compared to 2019 [63].

A summary of the main changes in transport related behaviour during COVID-19 restrictions across the Netherlands, Sweden and the UK is shown in Table 1.

**Table 1.** Summary of main changes recorded across the Netherlands, Sweden and the UK during COVID-19 restrictions*

	Netherlands	Sweden	UK
Percentage of people working from home	Increased from 37% to 66% between March 2020 and May–June 2020	Increased from 2% to 32% between February and April 2020	Increased from 11.4% to 36.2% before and during the first national lockdown (data collected May 2020)
Public transport use	From March 2020, use of public transport has decreased by 41–87% compared to the same week 2019	Between February and June 2020, a decrease of 40–60% in public transport use across was recorded across the three most populated regions in Sweden compared to the same time in 2019	Train use was down by 95% between March and May 2020 compared to the same period in 2019. Bus use was down between 80–85% decrease from March to June 2020 compared to January 2020
Private transport use	From March 2020 of those who would usually have used public transport, 41–60% opted for use of private cars to complete their journeys	A 10–30% decrease in car traffic during weekdays and a 30–40% decrease on weekends between March and May 2020 was observed	A 50–70% decrease in road traffic was recorded between March–May 2020
Active transport	From March 2020, of those who would usually have used public transport, between 14–57% used their bike and 17–38% walked to compete their journey	In April 2020, 28.6% of respondents reported walking and biking more since March 2020, mainly to avoid public transport	Cycling trips increased by 100–200% during April–June 2020

*Data is for illustration purposes only. Due to differences in data collection methods across each country, this table does not reflect direct comparisons.

## The likely impact of these transport and mobility behaviour changes on climate change

According to the International Energy Agency (IEA), there is the potential for 35% of the workforce in Europe to work from home long-term [26]. Other sources estimate a potential of 24–31% of home-based work in Europe [67]. The IEA estimates that the pre-COVID proportion of the workforce working from home in Europe was 5% [26]. The climate impact of working from home depends on several variables such as the nature of the job, country, residential energy use, commute length and mode of transport. The IEA estimates that if everybody who was able to work from home worldwide did so for 1 day per week, it could bring an annual decline of 24 million tonnes CO_2_ emissions (MtCO_2_) and would save 1% of global oil consumption for road passenger transport per year [26]. During the pandemic, it was estimated that 59% of the global workforce were working from home [26]. The reduction of surface transport accounted for just under half of the decrease in CO_2_ emissions in April 2020 compared to 2019 levels [3]. Decreased levels of journeys made by internal combustion engine vehicles as identified here across three countries can have a meaningful impact on carbon emissions. Fewer car journeys in rush hours imply decreasing traffic congestion leading to improved air quality. This change is consistent with the transport changes required for mitigation of climate change: ‘technical and behavioural mitigation measures for all transportation modes combined with new infrastructure and urban redevelopment could significantly reduce the energy demand of the sector’ [10]. As identified here, a decrease in commutes, increased active transport, and the use of electric vehicles accelerated during the COVID-19 pandemic and has the potential for significant climate mitigation effect if maintained [68]. Reasons for the decrease in internal combustion engine vehicle sales could be increased working from home, global shutdowns of the auto industry, disruptions to suppliers and showroom closures during COVID-19 restrictions. However, disruptions caused to sales of internal combustion engine vehicles caused by COVID-19 restrictions would also apply to the sale of electric vehicles. Additionally, there has been an increase in the trend of electric vehicle sales in globally recorded data in recent years, with the sales of plug-in hybrid electric vehicles and battery electric vehicles increasing by over 40% in 2019 compared to 2018 [26].

**Figure 1:**
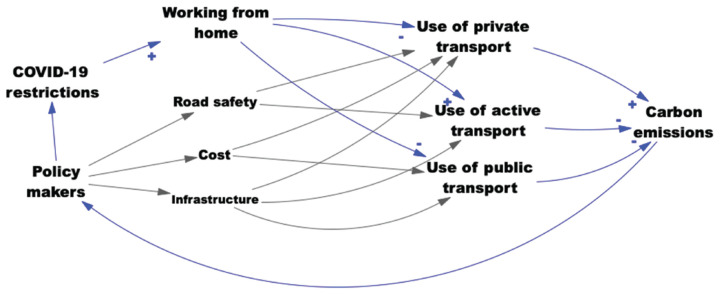
Working from home concept map. Arrows represent causal relationships, with blue arrows representing relationships illustrated by data. (Grey arrows represent variables and links that are known to be important but for which no data was sought for this commentary.)

To represent the impact of COVID-19 working from home guidance and other factors on transport use and carbon emissions, we developed a concept map using the available data, theory knowledge and expertise within the EBCN (see Fig. 1). In this map, arrows represent hypothesised directional causation. The ‘+’ or ‘-’ symbols represent the direction of change between the two variables linked by an arrow. For example, the ‘-’ signalled at the relationship between ‘working from home’ and ‘use of private transport’ shows that increases in working from home lead to an overall decrease in private transport use. This map represents an intervention and related actors (COVID-19 restrictions and policy-makers), the content of the system (working from home, use of private, active and public transport) and the impact of the intervention through the content on the variable (carbon emissions). This map shows the link between COVID-19 restrictions and increased working from home, leading to reduced use of private and public transport and increasing active transport, overall resulting in decreased carbon emissions. It indicates how removing the COVID-19 restrictions might impact carbon emissions.

## Insights from behavioural science relating to COVID-19 related behaviour change and potential impact on climate change

**Figure 2:**
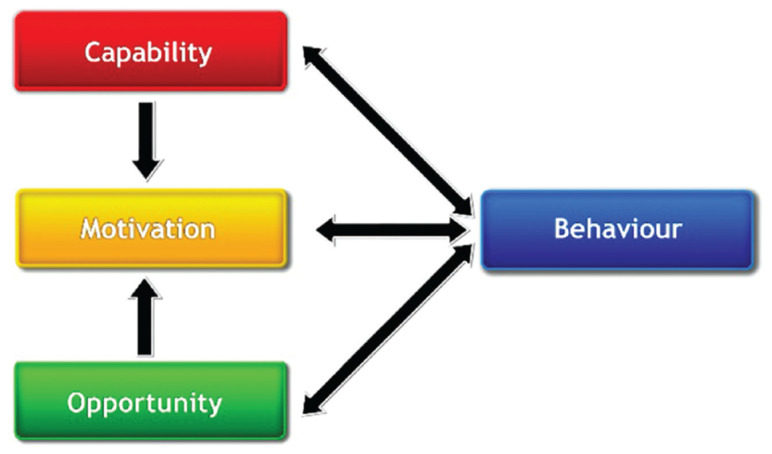
The COM-B model of behaviour [69]. COM-B, Opportunity, Motivation Behaviour (COM-B) model.

The Capability, Opportunity, Motivation Behaviour (COM-B) model [69] represents behaviour as an interacting system. Capability (physical and psychological skills required for a behaviour), opportunity (physical and social context needed for a behaviour) and motivation (brain processes required to direct and influence a behaviour) interact within this system to generate behaviour (see Fig. 2).

The COM-B model links to a framework of interventions and policies, the Behaviour Change Wheel, which in turn links to a taxonomy of 93 specific behaviour change techniques [14,69]. These tools have been used to inform the development and evaluation of interventions and policies concerning behaviour change in local government in England [70]. The Behaviour Change Wheel has also been used to illustrate the potential impact of behaviour change interventions to increase adherence to health-related policies during the pandemic [71] and to review pro-environmental behaviour change interventions [72–74]. Behaviour change theory can be used to hypothesise influences on identified behaviour changes that occurred during periods of COVID-19-related restrictions and guidance and to suggest ways to maintain behaviour changes likely to promote climate mitigation [1].

We cannot predict with certainty how citizens will behave once government recommendations and restrictions end as there is always uncertainty associated with predictions of future behavioural trends. However, based on COM-B we can predict that the behaviour changes most likely to be maintained are those where there are ongoing opportunities, capabilities and motivation to continue. The pandemic has led to changes in physical *opportunity* (e.g., lockdown policies and technologies enabling home working), improved *capabilities* to engage with digital home working options and increased *motivation* for working at home once restrictions end. Maintaining this pro-environmental behaviour change requires: ensuring that *opportunities* are sustained perhaps through policies relating to flexible working, *capabilities* are continually reviewed and improved to allow employees to connect with colleagues and maintain outputs, and strengthened *motivation*, for example, through interventions to improve employees well-being. Desires to return to pre-pandemic levels of consumption and travel, or beliefs that using public transport increases the risk of exposure to COVID-19, may increase reflective motivation to use private transport. Collecting and analysing data relating to transport behaviours will inform our understanding of if and how motivations, capabilities or opportunities change once government restrictions have ended.

The transport-related changes in behaviour we identified are strongly influenced by *opportunity*, with guidance and restrictions mainly advising against using public transport and avoiding travelling altogether, along with working from home recommendations and policies. Consequently, use of public transport decreased. There is evidence that *motivation* decreased for using public transport and increased for private transport use, due to understandings concerning how COVID-19 is transmitted and beliefs that public transport carries a high risk of COVID-19 infection [75,76]. *Opportunity* to use public transport could be increased by improved accessibility and infrastructure that allows for adequate spacing between passengers and other risk-reducing measures. Increasing *opportunity* may increase *motivation* by affirming beliefs that public transport is relatively safe, as would required behaviours of wearing face coverings and sanitising hands before and after transport use.

The identified shift in behaviour towards more pro-environmental active transport during the pandemic was also influenced by *opportunity.* Government restrictions on travel and local authority measures to implement active travel measures (e.g., ‘pop up’ cycle lanes, pedestrianisation, cycle hire schemes) to enable socially distant movement in towns and cities provided opportunities for active transport. Increases in various metrics of active transport during periods of government restrictions on travel, outlined in sections on Netherlands, Swedish and UK data, provide some evidence of the effectiveness of these measures. It is reasonable to assume that, in terms of COVID-19 transmission, active transport is believed to be low-risk. Levels of enjoyment of the experience of walking and cycling may also maintain or increase motivation.

The increase in levels of active transport behaviour is likely to have increased levels of physical *capability* relating to strength and stamina, along with psychological *capabilities* relating to cycling and navigation knowledge and skills. These changes present opportunities for policy relating to work and leisure to maintain capabilities while increasing opportunities for active transport through improving cycling and walking infrastructure. Introducing and maintaining *opportunity* for active transport within a working day would sustain or increase *capabilities* built on during the pandemic, along with potentially increasing pro-environmental-related *motivations* to maintain this identified behavioural change. *Opportunities* to purchase electric vehicles (including electric-bikes and electric-scooters) could be increased through financial incentives, hire schemes and the installation of charging points in areas not well connected by public transport [77]. Linking the purchase of electric vehicles to health and pro-environmental beliefs may also increase *motivation*.

The identified data suggest that overall private transport use decreased during COVID-19 restrictions [6,39,57,62]. However, self-report data indicates that intentions to make individual journeys using private transport increased during COVID-19 restrictions [43]. There is a risk that, if *capability, opportunity* and *motivations* for public and active transport decreases, this could lead to increases in private transport use when fewer restrictions are in place, with the impact being increased carbon emissions. Additionally, there is a possibility of long-term changes in urban structure and land uses attributable to COVID-19-related restrictions. These changes may result in people and companies moving away from city centres, decreasing *motivation* for investment in and use of public transport. Although changes in policies designed to maintain and increase public and active transport use may have a spill-over effect in decreasing private transport use, it will be necessary to implement specific discouraging actions, for example, with the use of low-traffic neighbourhoods, to reduce *opportunity* for private transport use, which may result in a decrease in *motivation* for using private transport. For instance, a reduction in car ownership was reported 2 years after introducing low-traffic neighbourhoods in Outer London [78].

From a behavioural perspective, to maintain behaviour changes influenced by this moment of change requires ensuring that *opportunities* are sustained or expanded, *capabilities* are continually reviewed and improved and *motivation* is strengthened. Additionally, reversing changes related to environmental cost requires removing *opportunities* or decreasing *motivation* for carbon-intensive behaviours. Increases in positive environmental behaviours were recorded in participants who had recently relocated compared to those who had not, suggesting that these were experienced as moments of change [79]. Evidence suggests that interventions increasing social and physical opportunities may be most effective, with some evidence for behaviour changes being maintained over time [74].

## Policy implications

To increase the likelihood of long-term behavioural changes to reduce climate change, building on behavioural changes identified during the disruption of the pandemic through policy is necessary. Government action and recovery-package incentives could have a significant long-term effect on global greenhouse gas levels [3], with the potential to avoid 0.3°C climate change by 2050 [23]. During the COVID-19 pandemic, we have observed that frequent emphasis on the desired behaviour of citizens by governments increases the likelihood of behaviour change [80]. We can link our behavioural analysis using the COM-B model to potential interventions and policies identified in the integrative framework, the Behaviour Change Wheel [69]. Persuasive and informative communication strategies, as well as environmental and social planning, will be key types of intervention and policy. As nations with more highly developed economies are known to produce the most carbon emissions [23], these nations are the ones that should take the most actions to mitigate carbon emission-related impact.

## Working from home

Employees working from home reduce their commuting-related behaviours. Many firms now offer workplace flexibility (e.g., Microsoft, Facebook and Salesforce), allowing employees the opportunity to choose in what form, and if at all, they return to working from the company’s offices [81]. Changing metrics of productivity, for example, by measuring outcomes instead of inputs, suggests that virtual workers deliver a high quality of work [82]. Policies that focus on remote or flexible work locations may benefit both employers and employees who wish to work in this way; communication strategies could draw attention to the benefits of working from home, or in local ‘hubs’, both for the employees and for the employers. Companies are likely to improve digitally mediated meeting and networking opportunities, and governments need to ensure good digital access for all. Monitoring the impact of working from home on employee well-being, along with improving and increasing opportunities for colleagues to interact socially, are important considerations for employers. Within Europe it is estimated that between 24% and 31% of the workforce has the potential to work from home, with pre-COVID levels of working from home estimated to be 5%. [26]. Therefore, although there remains a large proportion of the workforce across Europe whose work depends on travel, a significant proportion of the workforce may have the potential to work from home.

## Public transport

Increasing opportunities to use public transport through improved infrastructure will benefit citizens living within and outside cities. Improved infrastructure includes having sufficient volume, routes, timetables, bus sizes, ventilation, bus stops, traffic management and acceptable pricing. Improving communications regarding health and safety measures taken across public transport networks is likely to increase motivation to use public transport by affirming beliefs that public transport is relatively safe. Encouraging a gradual reintroduction of public transport use may help re-establish confidence, and good communications may be persuasive in terms of the environmental benefits of using public transport.

## Private transport

Intentions to use private vehicles more, after COVID-19 restrictions end, were identified in the Netherlands [34], and sales of electric vehicles increased in all three countries [45–52,58,63]. The increase in sales of electric vehicles was happening prior to, and in parallel with COVID-19-related restrictions, rather than because of them. However, policy-makers could use the moment of change represented by the pandemic to consider ways to support the use of private electric vehicles (including electric-bikes and electric-scooters) over internal combustion engine vehicles, to uphold motivation and increase opportunities to maintain the increase in electric vehicle sales. For instance, various stimulus packages have been launched across Europe to support purchasing an electric vehicle [83]. Investment and expansion of public charging outlets, along with accessible and transparent pricing models, is also required as this is an opportunity-related barrier to purchasing electric vehicles [77]. Further, policy-makers could consider how electric vehicles could be integrated into the public transport system by focusing on urban infrastructure, ensuring dedicated spaces and increasing opportunities and motivation to use these vehicles. Local policies relating to electric vehicles have emerged in several cities globally [84]. Increased use of electric vehicles could improve air quality and subsequently lower health risk as they have no tailpipe emissions and emit less heat [85]. The health and pro-environment-related factors may increase reflective motivation to continuing electric vehicles use on an individual level. Data collection regarding the impact of increasing sales of electric vehicles on transport-related carbon emissions will increase understanding of this behaviour.

## Active transport

Providing opportunities to increase or maintain bike use levels may be achieved by making pop-up bike lanes permanent, working with bike-sharing services to offer free ‘taster’ rides and increasing the number of bikes and electronic-bikes available. Increasing the availability and accessibility of spaces for active transport modes such as walking and cycling could have long-lasting health benefits by reducing the risks of cardiovascular disease, type-2 diabetes and mental distress [86]. Government and public health-related communication strategies could highlight and inform citizens about the potential personal benefits and frame active modes of transportation as healthy and good for the environment to motivate more citizens to adopt and maintain pro-environmental travel behaviours. The concept of local living or the 15/20-minute city concept, whereby people have access to education, shopping, employment and community facilities within 15/20 minutes of their home, is one option for achieving and maintaining high levels of active transport [87]. Local living promotes sustainability and liveability, along with improving the well-being, social and economic aspects of the lives of citizens. Local living allows citizens the opportunity to shift from a car-dependent urban structure to relying mainly on walking and cycling, significantly reducing carbon emissions [88]. Moving from concept to practice, the Netherlands and some parts of London have introduced low-traffic neighbourhoods where private motor vehicles can only access businesses and homes and cannot cross through neighbourhoods [89]. Evaluations of low-traffic neighbourhoods suggest links to increases in active travel through higher likelihoods of cycling [90].

## Future work

This commentary describes the impact of COVID-19-related regulations and restrictions on mobility and the potential for sustained climate mitigation across the Netherlands, Sweden and the UK. Further evidence, through surveys and interview-based studies conducted across countries, is needed to confirm trends identified in this commentary and extend analyses beyond work-related land-based travel to include aviation and shipping. Producing a standardised set of data would enable comparisons, highlighting similarities and differences between countries, and drawing potential links to cultural differences, trust in institutions and adherence to policy. Policy changes will have a differing impact on various sections of society. The behaviour changes identified within this report only relate to citizens who are employed. To ensure policies do not create or exacerbate existing social and economic inequality, analysis of socio-economic differences within and between nations are needed to identify those likely to benefit and those who may lose out due to long-term changes induced by the impacts of the COVID-19 pandemic. Economic modelling and scenarios could evaluate the mitigation potential of behavioural trends under different conditions, for example, a long-lasting global recession versus a rapid recovery or more ambitious climate commitments taken by governments. The long-term implications on changing behaviours due to the COVID-19 pandemic are unknown. Actions citizens are currently taking to mitigate the spread of COVID-19 could be built on to promote more climate action, including lobbying for governmental changes [1].

## Conclusion

COVID-19 represents a moment of change, during which citizens may be open to new information regarding pro-environmental behaviour changes. Opportunities relating to working from home introduced by COVID-19-related restrictions and regulations, improvements in capabilities to maintain work-related efficacy, and increases in motivation to work from home once restrictions and regulations end could lead to a decrease in carbon emissions through a reduction of commuter road traffic. To maximise the likelihood of maintaining potentially pro-environmental behaviour changes observed in 2020, policy changes, informed by behaviour science, are required. The intended outcomes of interventions to maintain pro-environmental behaviour changes show similarities across the three countries we examined. However, the different transport systems and work-related cultures mean that different strategies for implementing policies may be necessary. Inter-country networks who bring together expertise across different relevant disciplines, such as the European Behaviour Change Network, can enable understanding of how different interventions could be implemented across different geographical locations.

## Note

1 ’Electric vehicles’ were defined differently between the Netherlands, Sweden and the UK. Where it was available, we have included information regarding how electric vehicles were defined.

## Appendix 1 – Methods

### Data sources

We searched for freely available data (including but not restricted to: attitudinal surveys, sales data and transport ticketing data) from government, transport and commercial sources in the Netherlands, Sweden and the UK and sought reports and datasets through members of the EBCN and colleagues.

To have an accurate picture of the context in which any changes in behaviour were recorded, we also searched government websites for policy or guidance documents within the Netherlands, Sweden and the UK, requesting or legislating changes that would impact on behaviour, such as the closure of cafes and restaurants.

Differences were observed in the ways that data was collected between countries. We did not conduct quality assessments on the data we used, and no weighting was applied to any particular type or source of data. Datasets did not include identifiable information of individuals.

### Criteria for data inclusion

#### Population

Citizens living in the Netherlands, Sweden or the UK.

#### Language

English, Dutch or Swedish languages.

#### Outcomes

(i) Validated or industry-standard metrics, for example, total number of cars passing a particular point, during a specific time frame or (ii) self-reported behaviour.

#### Comparison

To illustrate differences in behaviour between time points, we included sources that had data from at least one-time point before COVID-19 restrictions began, and from within the timeframes of COVID-19 restrictions.

### Data extraction and analysis

Using a data extraction form developed for this study (Supplementary File 1), we extracted data on: country; region (international, national or regional); city (if applicable); population (general adult, etc.); time frame; main focus of source (car, walking, etc.); whether a change in behaviour change was recorded; brief description of behaviour change; likely impact on climate change (positive impact = lower carbon emissions); evidence of opportunity to maintain behaviour change (any reference to policy change, etc.) and evidence of motivation to maintain behaviour change (any evidence of attitude change, etc.).

Data from each country was extracted by a single author and its inclusion was reviewed by a subset of the authors.

### Data synthesis

A descriptive narrative synthesis of data was conducted for each research question for each country. A similar synthesis was conducted to explore similarities and differences between the countries. Where possible, differences due solely to legislation differences were highlighted. A concept map was developed using the available data, theory knowledge and EBCN expertise to visually represent influences on behaviour. We sought feedback from experts within the EBCN network and used this feedback to inform the final concept map.

## Appendix 2 – Data sources from each country

**Table A1.** Data sources for the Netherlands case

Data source	Outcome assessment	Behaviour change observed during COVID-19 pandemic (up to December 2020)
ANP 2020 [44]	Secondary analysis of data from car-sharing companies	Increase in use of car-sharing
Autoscout 24 2020 [53]	Self-report from sample of car users	Increases in intentions to use car
Bakker et al. 2020 [41]	Primary analysis of public transport data	Decrease in use of public transport
De Haas et al. 2020 [34]	Primary analysis of self-report data	Decrease in overall mobility
Kamphuis 2020 [43]	Primary analysis of self-report data	Decrease in intentions to use public transport
Koninklijke RAI Vereniging	Primary analysis of self-report data and sales data	Increase in proportion of people working from home; increase in intentions to use a car; increase in sales of electric vehicles
Royal RAI Association	Primary analysis of self-report data	Increased intentions to use private modes of transport when restrictions are lifted
Statistics Netherlands 2020 [42]	Primary analysis of public transport data	Decrease in use of public transport
Taale et al. [38,39]	Secondary analysis of self-report data	Decrease in use of public transport; increase in intention to cycle
Translink 2020 [40]	Primary analysis of public transport data	Decrease in use of public transport
Van Hagen and Ton 2020 [36]	Primary analysis of self-report data	Increase in intentions to work from home; increased intentions to avoid rush-hour travel; increased intentions to use car
VMS Insight 2020 [37]	Primary analysis of self-report data	Increases in proportion of people working from home; increases in intention to avoid rush-hour travel

**Table A2.** Data sources for the Swedish case

Data source	Outcome assessment	Behaviour change observed during COVID-19 pandemic (up to December 2020)
Åkerberg et al. 2020 [60]	Primary analysis of self-report data	Increases in walking and cycling
Bil Sweden 2020 [58]	Primary analysis of car sales	Increase in the proportion of e-mobility modes sold
Dahlberg et al. 2020 [56]	Primary analysis of GPS data from mobile phones	Decreases in overall mobility
Rumpler et al. 2020 [7]	Primary analysis of noise patterns	Decrease in levels of noise
SLB Analys 2020 [6]	Primary analysis of air particulates	Decrease in levels of particulates in air
Stockholm City 2020 [59]	Primary analysis of active transport flow	Decrease in proportion of people waking, changes in proportion of people cycling
Tele2 Sverige AB 2020 [54]	Primary analysis of self-report data	Increase in proportion of people working from home; increase in proportion of people with intention to work from home
The Swedish Confederation of Professional Employees	Primary analysis of self-report data	Increase in proportion of people with intention to work from home
Trafik Stockholm 2020 [57]	Primary analysis of traffic count	Decrease in overall traffic levels

**Table A3.** Data sources for the UK case

Data source	Outcome assessment	Behaviour change observed during COVID-19 pandemic (up to December 2020)
Citymapper 2020 [66]	Primary analysis of data from Citymapper users	Decrease in public transport use
Department for Transport 2020 [62]	Primary analysis of transport data	Decrease in road traffic; decrease in public transport use; Increase in cycle use
Fuller 2020 [4]	Secondary analysis of air quality	Decreases in levels of NO_2_ in air in London
Purple 2020 [64]	Primary analysis of Wi-Fi hotspot use	Decrease in public transport use
The Centre for Climate Change and Social Transformation 2020 [61]	Primary analysis of self-report data	Increase in proportion of people working from home; Increase in proportion of people with intention to work from home
The Society of Motor Manufacturers & Traders 2021 [63]	Primary analysis of vehicle sales	Decreases in vehicles sold; increase in e-mobility modes sold
Transport for London 2020 [65]	Primary analysis of transport use in London	Decrease in public transport use; increase in cycle use

## Appendix 3 – Restrictions and recommendations in The Netherlands, Sweden and the UK

### Political decisions in The Netherlands during COVID-19

The Dutch government used a mixture of restrictions and recommendations, referring to the recommendations as ‘basic rules’. These recommendations included working from home as much as possible, keeping 1.5-m distance from others, limits on the number of visitors at home and no unnecessary public transport travel. The list in Table A4 was based on sources from the Dutch government [91–100].

**Table A4.** List of recommendations and restrictions in The Netherlands during COVID-19

In effect (2020)	Recommendation or restriction
12 December	Closure of bars, restaurants and cafes
1 June–14 October	Bars, restaurants and cafes re-open, 30 guests were allowed to sit inside
24 March–11 May	First national lockdown
15 March–31 June	Gyms and other sport facilities closed
24 March–11 May	Hairdressers, nail salons and other ‘contact’ services closed
March, April and May and 4–18 November	Zoo’s, theatres, cinemas and other public locations closed
March–June	Public transport limit number of seats available for passenger use
16 March–June	Schools closed
March	Stores and markets must take measures to ensure social distancing between customers
March	Public events banned
March	Advised to work from home unless impossible
March	Limits on number of indoor visits to a household

### Political decisions in Sweden during COVID-19

The Swedish response to the pandemic in 2020 was unique compared to most other countries in the world, with less restrictive social distancing policies and recommendations rather than more drastic policies such as lockdowns. The response relied heavily on citizens taking responsibility and following the recommendations. In Sweden, The Public Health Agency (Folkhälsomyndigheten) is an expert agency with a role for informing the government and the general public on public health matters, and a responsibility to provide suggestions for public policies. Table A5 summarises recommendations and restrictions announced by the Public Health Agency of Sweden and the Swedish Government (Regeringskansliet) between March and December 2020 [101–122].

**Table A5.** List of recommendations and restrictions in Sweden during COVID-19

In effect 2020	Recommendation or restriction
12 March	Ban of public gatherings of more than 500 people
13 March	Recommendation for anyone with symptoms of common cold to stay home
16 March	Recommendation for remote work in Stockholm
16 March	Recommendation for anyone aged over 70 years of age to minimise physical interactions
17 March	Recommendation for remote teaching at high schools and universities, followed by a gradual closing of universities and upper secondary schools to students
19 March	Recommendation to avoid unnecessary travel
24 March	Restriction to allow table service only, in bars and restaurants
27 March	Banned public gatherings of more than 50 people
1 April	Recommendation to keep distance from each other, including recommendations for stores to limit number consumers, sport practice to be hold outdoors, limit number of travellers in public transport and adapt amount of public transport adapt to limit crowding
13 June	The recommendation to avoid unnecessary journeys is lifted
15 June	The recommendation on distance education for high schools is lifted
1 July	New law in effect stipulating restaurants have a responsibility to take measures to prevent the spread of COVID-19
30 July	Recommendation to keep working from home until autumn when possible
1 October	Recommendation to avoid visiting elderly home lifted in some cases
19 October	The FoHM decides to allow local authorities to enforce local recommendations and restrictions
20 October	Stricter social distancing recommendations in Uppsala län
22 October	The recommendations for anyone aged over 70 years of age to minimise physical interactions is lifted
27 October	Stricter social distancing recommendations in Skåne län
29 October	Stricter social distancing recommendations in Stockholms län, Västra Götalands län and Östergötlands län
3 November	Stricter social distancing recommendations in Jönköpings län, Hallands län and Örebro län
3 November	Limitation of the number of allowed people to be seated at the same table in restaurants to eight
5 November	Stricter social distancing recommendations in Kronobergs län and Södermanlands län
10 November	Stricter social distancing recommendations in Kalmar, Norrbotten and Västerbottens län
19 November	Stricter social distancing recommendations in Jämtlands län
20 November	Interdiction to serve alcohol after 22:00 pm
20 November	Interdiction to organize public gatherings and events with more than eight participants
20 November	Recommendations to plan to avoid crowding for Christmas shopping
1 December	Stricter social distancing recommendations in Blekinge län

### Political decisions in the UK during COVID-19

The UK Government used various restrictions to stop the spread of COVID-19. The main focus was on restricting people of different households mixing together by advising citizens to work from home and avoid the use of public transport unless necessary. There were also measures in place prohibiting inside and outside gatherings of people, at times these even included a ban on more than two people from different households meeting outside. It should be noted that while there were general instructions for all citizens of the UK, the governments of England, Scotland, Wales, and Northern Ireland all implemented different additional restrictions. Table A6 summarises the restrictions set out by the UK Government for the period March 2020 to December 2020.

**Table A6.** List of recommendations and restrictions in the UK during COVID-19

In effect (2020)	Recommendation or restriction
23 March–13 May	National lockdown
14 September	Gatherings of more than six people banned
18 September	Businesses selling food or drinks to make sure of appropriate distance between tables and not allowed to serve groups larger than six
September and October	Tier system in place
23 October–9 November	Wales goes into lockdown
4 November–2 December	National lockdown
2 December	Tier system in place
